# Neuronal Correlates of Cognitive Control during Gaming Revealed by Near-Infrared Spectroscopy

**DOI:** 10.1371/journal.pone.0134816

**Published:** 2015-08-05

**Authors:** Matthias Witte, Manuel Ninaus, Silvia Erika Kober, Christa Neuper, Guilherme Wood

**Affiliations:** 1 Department of Psychology, University of Graz, Universitaetsplatz 2, 8010 Graz, Austria; 2 BioTechMed Graz, Graz, Austria; 3 Laboratory of Brain-Computer Interfaces, Institute for Knowledge Discovery, Graz University of Technology, Inffeldgasse 12/IV, 8010 Graz, Austria; University of Montreal, CANADA

## Abstract

In everyday life we quickly build and maintain associations between stimuli and behavioral responses. This is governed by rules of varying complexity and past studies have identified an underlying fronto-parietal network involved in cognitive control processes. However, there is only limited knowledge about the neuronal activations during more natural settings like game playing. We thus assessed whether near-infrared spectroscopy recordings can reflect different demands on cognitive control during a simple game playing task. Sixteen healthy participants had to catch falling objects by pressing computer keys. These objects either fell randomly (RANDOM task), according to a known stimulus-response mapping applied by players (APPLY task) or according to a stimulus-response mapping that had to be learned (LEARN task). We found an increased change of oxygenated and deoxygenated hemoglobin during LEARN covering broad areas over right frontal, central and parietal cortex. Opposed to this, hemoglobin changes were less pronounced for RANDOM and APPLY. Along with the findings that fewer objects were caught during LEARN but stimulus-response mappings were successfully identified, we attribute the higher activations to an increased cognitive load when extracting an unknown mapping. This study therefore demonstrates a neuronal marker of cognitive control during gaming revealed by near-infrared spectroscopy recordings.

## Introduction

Goal-oriented behavior requires an orchestrated network of brain activations including sensory, motor and cognitive processes [1,2]. These processes have been summarized under the term ‘cognitive control’. For an effective control, our brain frequently has to link a given stimulus to an appropriate behavioral response. Stimulus-response mappings of this kind are therefore a central component of inductive reasoning [3] allowing for quick and adaptable human behavior.

There is an extensive literature on stimulus-response mappings that assessed how associations are formed and maintained [4]. This past research can be summarized under the term rule-guided behavior and has mostly relied on experiments using single responses to a given cue, for example in the Wisconsin Card Sorting Test [5] or in the Brixton Spatial Anticipation Test [6]. The neuronal populations involved in rule-guided behavior have been located in a distributed network across frontal, parietal and temporal brain regions [4,7,8]. Prefrontal cortex plays an important role [9] and especially the left dorsolateral prefrontal cortex (dlPFC) has been linked to the maintenance of rules [4,10]. Recent studies further suggested a specific regional organization. In particular, more complex rules involve progressively more anterior regions [7,11]. Yet, there is also growing evidence for a role of dlPFC in early phases of rule extraction and learning [12–14]. It thus seems that activations across brain areas may change along with the acquisition of a new rule. This idea was recently support by findings using a spatial rule attainment task [13] that demonstrated early dlPFC and frontopolar activations shifting to temporal and premotor regions when the rule was established. More general, a fronto-parietal control network has been linked to an initial adaptive mode of control [15,16].

Despite these insights coming from fMRI, only a few researchers applied event-related designs using electroencephalography. Mostly a late positive component has been described [17–20] reflecting either rule violation or hypothesis evaluation and generalization. Li et al. [21] showed that this positivity decreased from learning to application periods suggesting similar dynamics as revealed by the fMRI results mentioned above. However, the majority of experiments were based on artificial language grammar or arithmetical tasks. There is a lack of studies on naturalistic settings that monitor brain activations during continuous tasks. One exception is a recent study showing changes in theta power of electroencephalography after video game playing that resulted in enhanced performance in cognitive control tasks [22].

In the current approach, we therefore explored neuronal activity during game playing that involved simple stimulus-response mappings. We used a previous 2D game of ours [23] where participants had to catch falling objects. Those objects either fell randomly (RANDOM task), according to a known stimulus-response mapping based on color or shape (APPLY task), or according to an unknown mapping of the same type (LEARN task). We expected performance increases from RANDOM to LEARN to APPLY reflecting the different levels of task complexity (see Material and Methods for details). In addition, once a stimulus-response mapping is known and validated participants should perform better at catching the next objects falling. Near-infrared spectroscopy (NIRS) allowed exploring neuronal activity related to processes of cognitive control during gaming. NIRS is similar to fMRI in that it reflects the hemodynamic response in cerebral vessels [24–26]. The observed changes in hemoglobin concentration have been recorded in different movement paradigms [27,28] and can even represent activations during motor imagery [29,30]. Compared to most fMRI scanning protocols, NIRS offers a better time resolution [31,32] and is more mobile and robust to movement artifacts [33]. Prior studies have shown specific hemoglobin changes in prefrontal cortices during tasks of working memory [34–37] and video game playing [38,39]. We thus hypothesized that modulating the demands of cognitive control in a game task should be detectable in non-invasive NIRS signals over a network of different brain regions.

## Material and Methods

### Participants

Sixteen healthy adults (9 female, 7 male, mean age = 23 ± 2 years) enrolled in the current study after giving written informed consent. All of them were right handed, had no history of neurological or psychiatric disorders and had normal or corrected-to-normal vision. One additional participant was excluded from the analysis due to an increased amount of artifacts in the recorded brain activations. The study was approved by the local ethics committee (University of Graz) and is in accordance with the ethical standards of the Declaration of Helsinki.

### Gaming task

**Fig 1:**
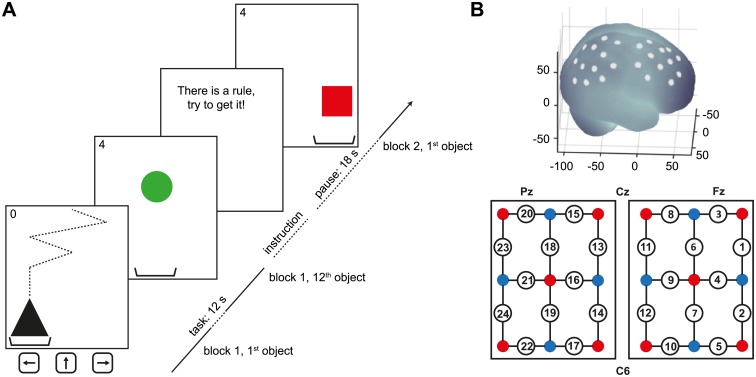
Experimental setup. (A) The 2D game displayed objects differing in color and shape on a computer screen. After an instruction, objects had to be caught by pressing the arrow keys associated with one of the three possible slots. A high-score in the upper left corner indicated the current performance. (B) Projections of the 24 channel positions (white points) on a MNI-152 compatible canonical brain. Two connected optode probe sets were positioned over the right hemisphe Similar to Fig 2 of the manuscript, these curves represent the amount of hemoglobin difference oxy-deoxy (delta Hb) in relation to the cumulative sum of objects successfully caught. This time each plot illustrates the grand average for our predefined ROIs (prefrontal: channels 1–5; sensorimotor: channels 11–14; parietal: channels 20–24). The rationale was to verify that the higher reactivity during the LEARN task can be found in each of these regions, as can be clearly corroborated here. In addition, one can observe an intermediate increase for activations of sensorimotor areas during the RANDOM task possibly reflecting the correlate of motor responses per se. Cost-benefit curves for the different ROIs.re according to the international 10/20 placement system. Red and blue circles indicate sensors and detectors, respectively. Numbers refer to the recorded NIRS channels.

We designed a simple 2-D game ‘U get it U catch it’ (Fig 1A; online information at http://studies.seriousgamessociety.org) in Matlab (The MathWorks, Natick, USA), where participants had to catch falling objects with a moveable paddle by pressing three predefined keys (arrow left, middle, right) of a conventional computer keyboard. Objects fell, one after each other including a 500 ms pause between objects, from top to bottom in 20 steps with a high speed of 50 ms/step. This fast pace was chosen based on pilot data to ensure that objects could only be caught with knowledge of the stimulus-response mappings described below. The path was created by interpolating a route of six basic steps composed of left, middle and right positions. For the last three steps the object did not change position to enable appropriate responses of the player. Objects differed in shape (circle, rectangle or triangle) and color (red, green or black).

To assess stimulus-response mappings of inductive reasoning, we implemented three experimental tasks. Each task was composed of 12 falling objects within a block and these sequences were repeated over 12 randomized blocks interleaving all experimental tasks (*n* = 36 blocks across tasks). Hence, participants had to switch between the different tasks so that motivation and attention could be kept high. During the RANDOM task objects fell randomly into the three slots. During the LEARN task objects fell according to an unknown but learnable regularity defined either by color or shape. These regularities were fixed one-to-one mappings, e.g. red objects will fall to the left slot, green to the middle and black to the right. They were tested in pilot experiments and became fully evident after three objects at earliest. After completion of each of these blocks, participants had to fill out a multiple-choice questionnaire asking for the presumed stimulus-response mapping. Finally, the APPLY task followed the same type of mappings but this time the regularity was explicitly stated before the beginning of the block (and thus not questioned afterwards). For example, the message on screen would read ‘Color defines the rule: red objects fall to the left, green objects to the middle and black objects to the right’. Similar messages were displayed for the other tasks: ‘There is no rule’ for RANDOM and ‘There is a rule: try to get it!’ for LEARN.

An experimental session started with three test blocks to ensure that all tasks were understood. Then, participants played the whole set of randomized 36 blocks with self-paced initiation of the next block within the sequence by button press (delay to start 18 s). The behavioral performance was statistically analyzed in a 3 x 3 x 3 repeated measures ANOVA including within-subject factors TASK (random vs. learn vs. apply), BLOCK (block 1 to 4 vs. block 5 to 8 vs. block 9 to12) and OBJECT (object 1 to 4 vs. object 5 to 8 vs. object 9 to12). Assessing performance changes over objects within a block allowed us to monitor specific effects of the different tasks at hand. In addition, changes over blocks can reveal general effects over the whole task period, i.e. whether participants improve due to repetition or suffer from fatigue. Post-hoc t-tests were applied (Bonferroni correction) and all statistics considered a type I error of 0.05.

### NIRS recording and analyses

We assessed changes of hemoglobin using a continuous wave system (ETG-4000, Hitachi Medical Co., Japan; for details see [30]. To this end, two 3 x 3 optode probe sets (24 channels, 3 cm inter-optode distance) were mounted over right cortices, aligned to the central point Cz of to the international 10/20 placement system (Fig 1B). The rationale for this setup was that we wanted to reduce influences of the right moving hand and instead targeted neuronal correlates of the cognitive processes during gaming. The three-dimensional coordinates of the NIRS channels were mapped onto MNI space using ELPOS (zebris Medical GmbH) and brain areas were identified based on the 1988 Talairach Atlas (Table 1; for details see [30]). NIRS signals were sampled at 10 Hz and stored on a conventional computer.

**Table 1:** Anatomic labeling.

**Channel**	**Brodmann area**	**Description**
**1,4,7**	8,9	Includes FEF, DLPFC
**2**	9,10	DLPFC, FPA
**3,6,9**	6,8	PMC and SMA, includes FEF
**5**	10,46	FPA, DLPFC
**8,11**	6	PMC and SMA
**10**	6,8,9	PMC and SMA, includes FEF, DLPFC
**12**	4,6	MI, PMC and SMA
**13**	3,4,6	SI,MI, PMC and SMA
**14**	1,2,40	SI, supramarginal gyrus (Wernicke's area)
**15,18**	5,7	Somatosensory association cortex
**16**	2,5,40	SI, somatosensory assocation cortex, supramarginal gyrus (Wernicke's area)
**17**	40	Supramarginal gyrus (Wernicke's area)
**19,22**	39,40	Angular gyrus, supramarginal gyrus (Wernicke's area)
**20**	7	Somatosensory association cortex
**21**	7,19,39	Somatosensory association cortex, V3, angular gyrus (Wernicke's area)
**23**	7,19	Somatosensory association cortex, V3
**24**	19	V3

Abbreviations: DLPFC dorsolateral prefrontal cortex; FEF frontal eye field; FPA frontopolar area; MI primary motor cortex; PMC premotor cortex; SMA supplementary motor area; SI primary sensory cortex; V3 tertiary visual cortex

For offline analyses, we explored relative concentration changes (in m(mol/l) x mm) of oxygenated (oxy-Hb) and deoxygenated hemoglobin (deoxy-Hb) using custom-written Matlab routines. Raw signals were semi-automatically cleared from artifacts (criterion: amplitude of Hb-signal >+/− 3 SD) and filtered in a range 0.01 to 0.9 Hz (zero phase-lag Butterworth). Next, task-related activations were obtained by referring time courses during gaming (0 to 12 s) to a pre-task baseline interval (-5 to 0 s).

NIRS activations in motor tasks have revealed a distinct increase of oxy-Hb and concurrent decrease of deoxy-Hb [27,40]. Moreover, the difference between oxy-and deoxy-Hb is an adequate marker of cerebral blood flow [41,42]. We therefore quantified this difference for each channel and defined three regions of interest (ROI): a prefrontal region (channels 1 to 5), a central region (channels 11 to 14) and a posterior parietal region (channels 20 to 24). The rationale was that these ROIs have a prominent role in associative response behavior [4,13] while the selected channel locations in our setup are still distant enough to avoid potential activation overlap.

Differences between the experimental tasks in these ROIs were statistically analyzed in a 3 x 3 repeated measures ANOVA including within-subject factors TASK (random vs. learn vs. apply) and ROI (prefrontal vs. central vs. parietal). Difference-Hb was assessed as mean activations in the time window 6 to 12 s because of the commonly observed time-lag in movement-related NIRS studies [27].

To compare the neuronal activations across tasks, similarity analyses have been suggested [43,44]. These techniques are helpful to transform multivariate patterns into similarity matrices that allow inference of the relationships among the data. We applied multidimensional scaling that has been often used in cognitive psychology for data visualization [45,46]. In particular, we used the smacof (scaling by majorizing a complicated function) algorithm [47] with maximal *n* = 1000 iterations and a random start configuration. The input data were defined as the pairwise correlations between all channels in a time window 6 to 12 seconds. The resulting two-dimensional space represents the distances between the input channels in a way to best approximate their given dissimilarities, i.e. one minus the correlation coefficients.

## Results

### Behavioral performance

We first assessed the percentage of objects successfully caught by each participant to evaluate the gaming performance. In general, the game was hard to play so that participants succeeded in 35% on average. Statistical testing of the dependent variable 'objects caught' in an ANOVA revealed significant main effects for all within-subject factors TASK (*F*(1,15) = 9.31, *p* < 0.001, *η2* = 0.40), BLOCK (*F*(1,15) = 6.90, *p* < 0.01, *η2* = 0.33) and OBJECT (*F*(1,15) = 21.50, *p* < 1e-5, *η2* = 0.61). Participants caught more objects during the APPLY task (*M* = 40, *SE* = 7%) when compared to LEARN (*M* = 32, *SE* = 6%) or RANDOM (*M* = 33, *SE* = 2%) indicating that pre-known regularities improved the performance. They also were more successful in the middle (*M* = 36, *SE* = 3%) and last block (*M* = 36, *SE* = 5%) than in the first block (*M* = 33, *SE* = 3%) and caught more of the last four objects (*M* = 40, *SE* = 6%) when compared to the first (*M* = 32, *SE* = 4%) and middle part (*M* = 33, *SE* = 4%) of falling objects. These overall results could suggest an improvement both due to task-specific effects within blocks and due to general repetition effects over longer periods across blocks. The significant interaction TASK x BLOCK (*F*(1,15) = 3.29, *p* < 0.05, *η2* = 0.19) and an interaction TASK x OBJECT (*F*(1,15) = 16.47, *p* < 1e-5, *η2* = 0.54) allowed to further clarify the different improvements. Post-hoc tests (see S1 Table) indicated that only for the APPLY task more objects were caught in the last block compared to the first block. In addition, the last objects within a block were caught more often than the middle part. Both of these improvements point towards familiarization effects that will be discussed below. Most importantly, participants caught more of the last objects within a block when compared to the first and middle part of falling objects during LEARN suggesting that the stimulus-response mappings were correctly identified in the course of a given block. As expected, no significant differences were revealed for the RANDOM task: due to random stimulus-response mappings and the fast pace of falling objects, participants were not able to improve their performance.

The multiple-choice questionnaires filled out after each LEARN block indicated that across participants 88 ± 8% of the regularities were correctly identified. There was no effect of the type of mapping, i.e. color was as informative as shape (*t*(15) = -1.18, *p* = 0.26).

### Brain activations revealed by NIRS

**Fig 2:**
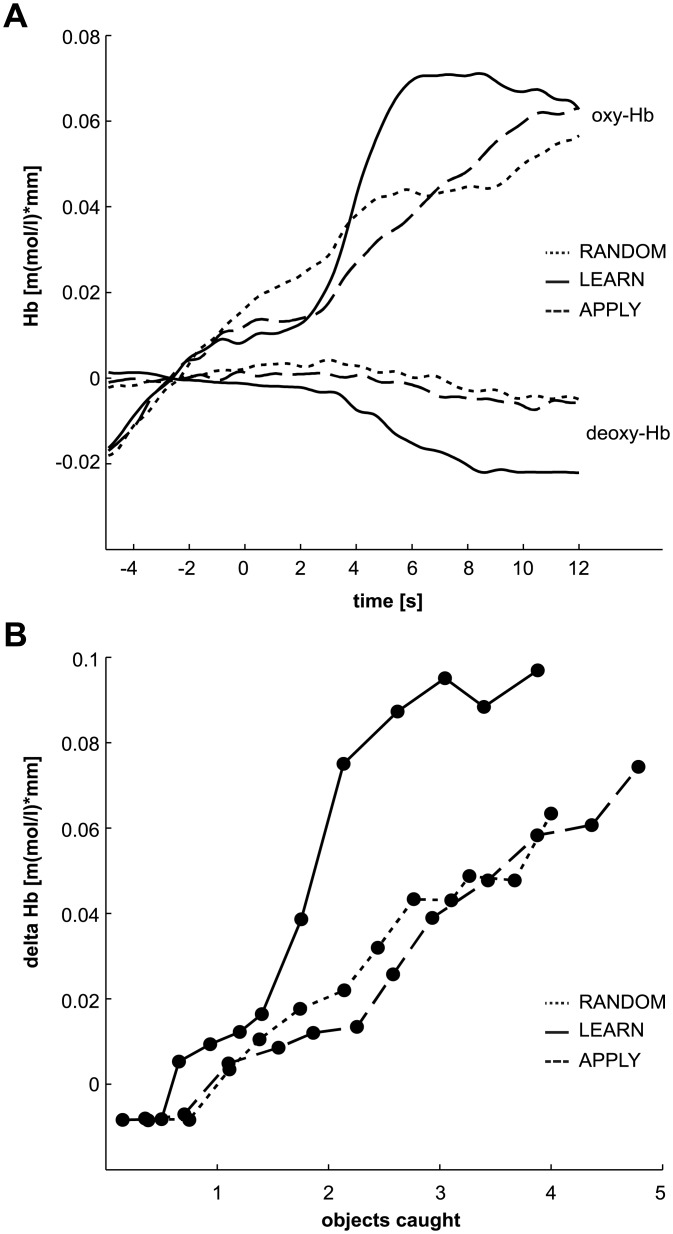
Average hemoglobin changes. (A) Changes of oxygenated (oxy) and deoxygenated (deoxy) hemoglobin (Hb) relative to a five second baseline for the three different tasks. The time courses represent a grand average over all channels and participants. Time point zero indicates the beginning of the game task. (B) Cost-benefit curves relating the grand average (over channels and participants) of the hemoglobin difference oxy-deoxy (delta Hb) to the cumulative number of objects caught. Each dot represents one of the twelve falling objects within a block. Note that delta Hb time series have been resampled.

The most consistent pattern of changes in NIRS signal across participants and channels was an increase in relative oxy-Hb concentration along with a decreased deoxy-Hb level (Fig 2A). On a descriptive level, a higher reactivity could be observed for the LEARN task starting from approximately 3 seconds of the task interval. Oxy-Hb peaked around 6 to 8 seconds on average, while deoxy-Hb reached a maximal decrease starting from 8 seconds on. In contrast, the RANDOM and APPLY tasks were associated with a less pronounced and gradual signal change for oxy-Hb and almost no change for deoxy-Hb.

To better quantify these changes, we analyzed the difference between oxy- and deoxy-Hb (time window 6 to 12 s). This difference index has been shown to reflect cerebral blood flow and is often used in tasks involving motor responses [27,40]. A 3 x 3 repeated measures ANOVA indicated significant main effects for both within-subject factors TASK (*F*(1,15) = 4.29, *p* < 0.05, *η2* = 0.22) and ROI (*F*(1,15) = 3.32, *p* < 0.05, *η2* = 0.18). In particular, Hb-differences were higher during LEARN (*M* = 0.082, *SE* = 0.014 m(mol/l) x mm) when compared to RANDOM (*M* = 0.048, *SE* = 0.015) and APPLY (*M* = 0.043, *SE* = 0.008). The main effect ROI showed higher signal change over central sensorimotor area (*M* = 0.071, *SE* = 0.009) compared to the prefrontal ROI (*M* = 0.047, *SE* = 0.011). However, the interaction TASK x ROI was not significant (*F*(1,15) = 1.06, *p* = 0.38).

In a ‘cost-benefit analysis’ we then sought to combine the behavioral and physiological data and to assess the overall cognitive load during the task. To this end, we calculated the cumulative sum of objects caught within a block against the Hb-difference. This index thus shows the neuronal effort associated with each of the falling objects. Fig 2B reveals that an overall increased neuronal activation was necessary during the LEARN task to catch the falling objects. In particular, a steep increase of the curve after the sixth object could be observed that saturated for the last three objects only. In contrast, the load profile for the remaining two tasks was similar and showed a gradually increasing activation. This overall pattern of higher reactivity was evident for all three selected ROIs (S1 Fig).

**Fig 3:**
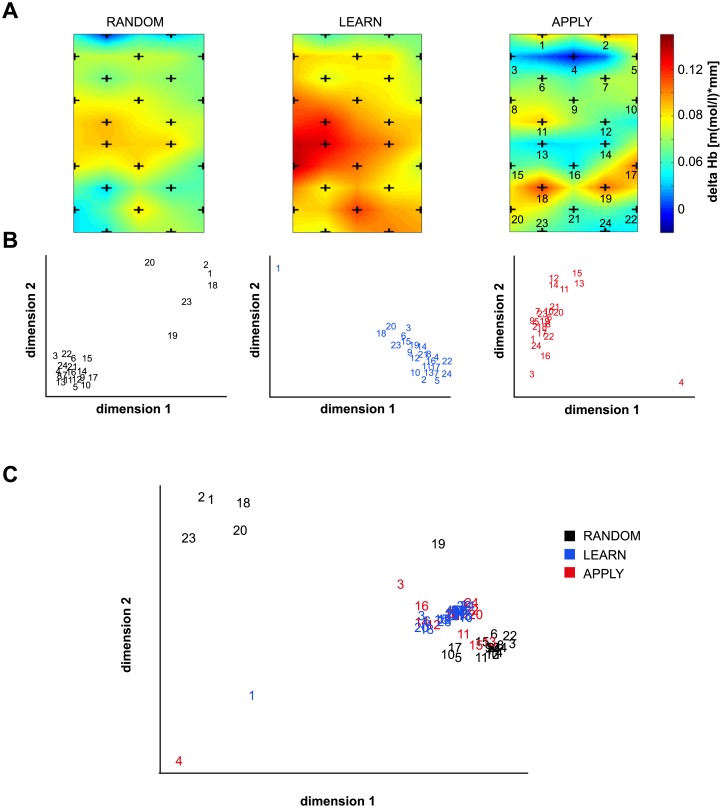
Topographical patterns. (A) Grand average topographies for the time period 6 to 12 seconds. Numbers indicate channels as shown in Fig 1. (B) Mapping of the activations (numbers again represent channels) to a two-dimensional space by multidimensional scaling. We used pairwise correlation between channels as input to an iterative procedure based on the smacof algorithm (for details see Methods). Channels with a high correlation will cluster together, thus revealing the structure of the whole network (stress values for RANDOM, LEARN and APPLY: 1.3, 1.0 and 0.6). (C) Same data as in (B) mapped into a common space (stress value: 0.003). Note that the resulting configuration dimensions are without a unit and lower stress values indicate a better fit.

Topographical plots of the average Hb-differences described statistically above corroborated an increased activation during LEARN over a broad cortical region (Fig 3A). The main focus was located over central sites extending to parietal and prefrontal regions. Similar albeit weaker changes were found for the RANDOM task, with a focus over primary sensorimotor areas. In contrast, the maximum activations during the APPLY task were mostly confined to posterior parietal and dorsolateral prefrontal areas. Because these average activation plots can only represent a first hint on the main areas involved, multidimensional scaling was used to visualize the different networks both within and across tasks according to their neuronal similarity [44]. We decided to use correlation over time as an index of functional connectivity here. Similar approaches have been applied for example to identify language networks during speech comprehension [48]. Multidimensional scaling optimized the positions of single channels in a low-dimensional space to represent the overall ‘similarity structure’ of a higher dimensional space [49]. Fig 3B displays the resulting two-dimensional coordinates that represent the similarities based on the correlation between pairs of channels. The LEARN task was associated with basically one extended cluster. In contrast, two separate clusters covering mostly pre- and post-central or fronto-parietal regions were identified for the RANDOM task. For the APPLY task, channels over primary sensorimotor areas were grouped together and slightly stood out from the remaining channels. Within this second larger cluster, a subset of fronto-parietal channels (channels 1, 3, 16, 24) could be observed. In addition to these clusters within a given task, we also pooled the correlation matrices of all three tasks as input to multidimensional scaling. Besides the identification of fine patterns, this provided a global comparison of the connectivity patterns across tasks (Fig 3C). The analysis revealed a high similarity between the LEARN and APPLY tasks, while the RANDOM task showed an overall different configuration.

## Discussion

In this study, we showed that different demands on cognitive control during gaming are reflected in changes of hemoglobin concentrations recorded with NIRS. An increased reactivity of NIRS signals over distributed areas including prefrontal, central and parietal brain regions could be observed when participants were required to extract simple stimulus-response mappings.

### Gaming performance

Our gaming approach was designed to put high demands on players so that successful performance could only be reached when participants were able to infer and implement the correct stimulus-response mappings. This is confirmed by the finding that during APPLY success rates were clearly above chance. In this task, there was an improvement for the last block and the objects falling latest within a given block. Although the correct mapping was known in advance, this may indicate that participants were uncertain about these regularities in the beginning and needed some time to get used to the task. During RANDOM we observed chance-level performance and no significant trends over repetitions and objects. This most likely reflects the intended reaction task depending on fast motor responses only. In contrast, participants were more successful catching late objects within a block for the LEARN task. As the overall performance was not different from RANDOM and no trend over blocks was detectable, we attribute this behavior to the cognitive demands associated with extraction of the stimulus-response mappings: only from the third object on, a given mapping can be inferred and subsequently has to be validated and applied. This process has to be repeated for each new block and likely impedes a better performance in the brief high-speed game. The unexpected chance-level performance during LEARN therefore might reflect the highly demanding settings of the game. At the same time, these settings allowed avoiding learning effects due to mere task repetition and prevented participants from catching objects without extraction of the mappings. Moreover, most stimulus-response mappings were identified in the post-hoc questionnaires so that participants most likely focused on extraction of these regularities rather than catching the falling objects. Future studies with more objects falling within a block could help to assess the transition from extraction to application of a given mapping. In this line, past work [7] indicated that one-to-one mappings between stimulus and response result in a learning curve with gradually increasing performance taking around 90 trials to exceed the lower confidence bound.

### Up-regulation of NIRS during gaming

Having shown behavioral differences, we next assessed the correlates in NIRS signals. The most distinct increase of reactivity for oxy- and deoxy-Hb was found only during the LEARN task. Further, the steep increase in oxy-Hb coincided with the time period when the hidden stimulus-response mappings could be discovered. As also illustrated by the cost-benefit curve, these results suggest an increased cognitive load when participants were required to extract unknown stimulus-response mappings. This is in line with past findings that have identified a network of brain areas specifically activated during initiation of cognitive control processes [15,16]. This network is supposed to respond to onset cues of the task and monitor feedback information during task performance. As required in our gaming task, higher activations of the associated brain areas can thus ensure a fast and adaptable cognitive control from trial to trial.

An alternative account for higher activations to consider is the role of motor processing. Previous reports have shown an increase of oxy-Hb during tasks of motor learning along with a decrease in deoxy-Hb [27,40]. These changes usually occur over a broad area around motor cortex [28]. Yet, participants did not catch more objects during LEARN when compared to the RANDOM task but still showed a higher level of activation. We also assessed NIRS signals ipsilateral to the moving hand, hence limiting pure motoric effects. This is supported by a study on finger tapping [28] that provided evidence for a bilateral change in oxy-Hb but localized deoxy-Hb changes in contralateral cortices only. Altogether, an increased cortical activation based on motor learning is thus not plausible in our view.

It may also be the case that simply holding a given instruction or regularity in memory results in increased cortical activation. Similar changes of hemoglobin have indeed been demonstrated during working memory tasks in monkeys [35] and humans [50], localized to prefrontal and parietal brain regions. Yet, the neuronal activation during LEARN is unlikely to reflect a mere increase of working memory load since during APPLY participants also had to remember and implement a pre-known stimulus-response mapping. This most likely resulted in demands similar to the learning task, especially because objects were falling at a fast pace. In summary, our findings suggest that NIRS signals during LEARN did not reflect memory processes alone but rather mirrored the level of cognitive control needed during gaming.

### Regional specificity of NIRS signals

The topographical analysis showed an extended network of up-regulated brain areas during the LEARN task. This network included regions from dorsolateral prefrontal to sensorimotor to parietal cortices (see Table 1). The significant effect of TASK along with a missing interaction TASK x ROI further supports this overall up-regulation. Moreover, multidimensional scaling revealed a single cluster of NIRS channels. Because this analysis considered correlations over time, one can assume a consistently activated network. In line with our results, Badre and colleagues [7] showed a high activation of premotor and prefrontal regions during the initial learning of simple stimulus-response associations. These activations only decreased after 120 trials. As our experimental design assessed short time periods, it thus seems plausible that a broad overlapping network was activated.

How can one interpret the high signal change over sensorimotor areas during LEARN in relation to existing literature on inductive reasoning? Most importantly, this pattern could reflect the type of task used: while other studies have relied on single button presses after a block of fMRI recording [12–14], we extended these paradigms to a fast, ongoing game design. As a consequence, this naturalistic design required more sensorimotor processing. Yet we did not find pronounced sensorimotor activation during the APPLY task but instead prefrontal (including frontopolar areas) and parietal regions were activated. Still participants caught more objects supporting the idea that they were able to maintain and implement the given stimulus-response mappings with a minimum of movement-related activity. Interestingly, the correlations between channels during LEARN showed a high similarity to the APPLY but not to the RANDOM task. Hence, across time a similar network of brain regions was activated yet to a different extent. This also suggests that in addition to mean activations it is worthwhile to assess the temporal dynamics more closely in future NIRS studies.

The activations during LEARN thus rather reflect the requirements of cognitive control. In particular, brain regions within prefrontal and parietal cortices have been linked to the decoding of cue information and appropriate response adjustment [16]. According to these authors a second network of cinguloopercular regions, including anterior prefrontal and anterior cingulate cortices, is responsible for maintaining task sets across longer time periods. Our experimental design fits well with the first component of this dual-network model of control. In addition, task complexity may have also played a role here as there is growing evidence for a rostro-caudal axis [7,11,13] with representations of less complex rules located more posterior. The non-hierarchical stimulus-response mappings of our task might thus contribute to the observed pattern of more posterior activation.

### Processes of cognitive control during gaming

At this point of our research we can only speculate about the details of the underlying processes in the gaming task. One can expect correlates of working memory, spatial attention, executive functions and general sensorimotor processing, all of which are best summarized under the term cognitive control. It has been reported that inductive reasoning involves a series of different steps [3,13]. Instances have to be collected and kept in memory to allow monitoring of specific stimulus-response mappings. Then regularities are identified that lead to generation of a hypothesis about the expected mappings. Expectations may be integrated with previous experiences and finally will result in a generalized rule. Finally, this rule is tested and applied in the subsequent instances.

These single processes can be considered a continuum. For example, Crescentini and colleagues [13] demonstrated prolonged activation of dlPFC and parietal regions during a spatial anticipation task. They concluded that alternative hypotheses are generated and maintained in a pending state. Moreover, in a modified version of the Wisconsin card sorting test that involved button presses instead of verbalizations, activations due to working memory and rule extraction overlapped [14]. This was especially pronounced for the right dlPFC. Interestingly, rule complexity did not modulate the strength of activation in this region [13]. This indicates a more general involvement in inductive reasoning in addition to the postulated specific role for the left dlPFC [8,10,14]. The increased hemoglobin reactivity over premotor and prefrontal regions during our LEARN task would in this sense allow monitoring alternative hypotheses [51] and selecting the most appropriate motor responses according to the expected stimulus-response mapping [4,13]. As mentioned above, this flexible initiation and adjustment of control in response to feedback is in line with a recent model of cognitive control [15,16].

All our results from behavioral and neurophysiological analyses indicate that more neuronal resources were allocated to fulfill our task requirements, i.e. extracting the unknown stimulus-response mapping and catching objects. This may also involve visuospatial attention during goal-oriented behavior [52,53]. Findings of increased evoked-potentials during discovery of regularities have also reported a fronto-central topography extending to parietal areas [21]. These authors suggested processes of attention towards the detection of regularities in a sequence of Arabic numbers. Due to the sequential nature of our task, similar effects may indeed have played a role. The fronto-parietal sub-clusters we found during both RANDOM and APPLY tasks also fit into this view. In addition, these regions could indicate processes of error detection and uncertainty: participants may have tried to create and validate stimulus-response mappings although they were not instructed to do so.

## Conclusions

We extended past work on inductive reasoning to a simple 2D game and demonstrated the neuronal correlates in NIRS recordings. So far, only few studies exist that report changed activation in prefrontal regions during gaming [38,54]. Moreover, there is some evidence for altered fMRI patterns in online gaming addiction [55–57]. Our preliminary data support the hypothesis that neurophysiological signals can serve as metric indexing the progress of learning [23]. Prolonged NIRS recordings during gaming might thus advance the field of serious games and learning analytics. Moreover, recent evidences suggest that training cognitive control in video games can result in generalized improvements in various untrained cognitive aspects [22,58]. Along with reports that correlates of cognitive control are stable across time and tasks [2], our results thus encourage further exploration of video game training in rehabilitation.

## Supporting Information

S1 Fig Cost-benefit curves for the different ROIs. Similar to Fig 2 of the manuscript, these curves represent the amount of hemoglobin difference oxy-deoxy (delta Hb) in relation to the cumulative sum of objects successfully caught. This time each plot illustrates the grand average for our predefined ROIs (prefrontal: channels 1–5; sensorimotor: channels 11–14; parietal: channels 20–24). The rationale was to verify that the higher reactivity during the LEARN task can be found in each of these regions, as can be clearly corroborated here. In addition, one can observe an intermediate increase for activations of sensorimotor areas during the RANDOM task possibly reflecting the correlate of motor responses per se.
(EPS)

S1 Table Comparison of number of objects caught. (DOCX)
