# Supplementary material for: Neuronal Correlates of Cognitive Control during Gaming Revealed by Near-Infrared Spectroscopy
Source: PLoS One. 2015 Aug 5;10(8):e0134816. doi: 10.1371/journal.pone.0134816 (PMC4526694; doi:10.1371/journal.pone.0134816)
Supplement: S1 Table — (DOCX) [file pone.0134816.s002.docx]

**S2 Table. Comparison of number of objects caught**

| Factor(s) | mean ± SD: | Level |  | Comparison | Post-hoc *p*-value |
| --- | --- | --- | --- | --- | --- |
|  | **1** | **2** | **3** |  |  |
| Task | R  4.0 ± 0.27 | Le 3.88 ± 0.77 | A 4.78 ± 0.82 | 1 vs 2  1 vs 3  2 vs 3 | 1.0  .0035  .0007 |
| Block | E 1.31 ± 0.05 | M 1.44 ± 0.04 | L 1.46 ± 0.05 | 1 vs 2  1 vs 3  2 vs 3 | .0186  .0104  1.0 |
| Object | E 1.29 ± 0.04 | M 1.31 ± 0.04 | L 1.60 ± 0.06 | 1 vs 2  1 vs 3  2 vs 3 | 1.0  .0000  .0000 |
| Task x Block | R x E 1.28 ± 0.05 | R x M 1.30 ± 0.04 | R x La 1.43 ± 0.03 | 1 vs 2  1 vs 3  2 vs 3 | 1.0  1.0  1.0 |
|  | Le x E 1.21 ± 0.09 | Le x M 1.43 ± 0.08 | Le x La 1.23 ± 0.07 | 1 vs 2  1 vs 3  2 vs 3 | .1833  1.0  .3889 |
|  | A x E 1.44 ± 0.09 | A x M 1.60 ± 0.07 | A x La 1.70 ± 0.09 | 1 vs 2  1 vs 3  2 vs 3 | 1.0  .0289  1.0 |
| Task x Object | R x E 1.38 ± 0.03 | R x M 1.39 ± 0.05 | R x La 1.23 ± 0.05 | 1 vs 2  1 vs 3  2 vs 3 | 1.0  1.0  1.0 |
|  | Le x E 0.94 ± 0.06 | Le x M 1.20 ± 0.09 | Le x La 1.75 ± 0.10 | 1 vs 2  1 vs 3  2 vs 3 | .2228  .0000  .0000 |
|  | A x E 1.56 ± 0.10 | A x M 1.36 ± 0.10 | A x La 1.83 ± 0.08 | 1 vs 2  1 vs 3  2 vs 3 | 1.0  .1458  .0000 |

Abbreviations: A Apply; E Early; La Late; Le Learn; M Middle; R Random
